# Carbothermal Reduction Synthesis of Aluminum Nitride from Al(OH)_3_/C/PVB Slurries Prepared by Three-Roll Mixing

**DOI:** 10.3390/ma14061386

**Published:** 2021-03-12

**Authors:** Qian Wen, Peng Wang, Jingwu Zheng, Yao Ying, Jing Yu, Wangchang Li, Shenglei Che, Liang Qiao

**Affiliations:** 1 College of Materials Science and Engineering, Zhejiang University of Technology, Hangzhou 310014, China; wenqian0814@126.com (Q.W.); wp15757172205@126.com (P.W.); zhengjw@zjut.edu.cn (J.Z.); yying@zjut.edu.cn (Y.Y.); yujing@zjut.edu.cn (J.Y.); wcli@zjut.edu.cn (W.L.); cheshenglei@zjut.edu.cn (S.C.); 2 Research Center of Magnetic and Electronic Materials, Zhejiang University of Technology, Hangzhou 310014, China

**Keywords:** aluminum nitride, polyvinyl butyral (PVB), Al(OH)_3_, carbothermal reduction–nitridation

## Abstract

Polyvinyl butyral (PVB) was used in the Al(OH)_3_/carbon black/ethanol slurries by the three-roll mixing to prepare AlN powder using the carbothermal reduction–nitridation (CRN) process in the experiments. The effects of PVB addition on the synthesis of AlN powder were studied by viscosity, tap density, XRD, SEM and TG measurements. The results showed that the PVB layer covering on the surface of Al(OH)_3_ particles reduced the viscosity of Al(OH)_3_/carbon/ethanol slurry and increased the dispersion homogeneity of Al(OH)_3_/carbon raw powder. The tap densities of the Al(OH)_3_/carbon mixtures after three-roll milling could be increased with the increase in PVB addition. In the CRN process, most of the PVB covering Al(OH)_3_ particles evaporated and supplied the passage for nitrogen removal to the particles. Based on the experimental data, the role of PVB on the mixing and CRN process was discussed.

## 1. Introduction

In the past twenty years, increasing attention has been paid to aluminum nitride (AlN) including AlN thin films, ceramics and composites due to high thermal conductivity, high electrical resistivity, good electrical insulation, non-toxicity and thermal expansion coefficient close to that of silicon [1,2]. AlN thin films with high band gap (~6.2 eV), acoustic velocity and a substantial electromechanical coupling coefficient were used for surface acoustic wave (SAW), bulk acoustic wave (BAW) and heterojunction diodes devices in piezoelectric and semiconducting fields [3,4,5]. Using AlN powder as raw material, AlN ceramics with high thermal conductivity (>170 W/(m⋅K)), low dielectric constant (~8.8) and loss (~10^−4^) could be prepared by tape casting and sintering, which were usually used for substrate material of large-scale integrated circuit and high-power packaging of light-emitting diodes (LED) in electronic and optoelectronic fields [1,6]. In addition, AlN powder with good distribution and spherical shape was also used as a filler for thermal conductive composites, which was a promising packaging material [7,8].

In order to obtain the high-performance AlN ceramics, it was necessary to prepare AlN powder with good properties. Comparatively, the carbothermal reduction–nitridation (CRN) process had more advantages for AlN powder with high purity, high stability against humidity and high sinterability [9,10]. The difficult mixing of fine Al_2_O_3_ and carbon black powders, high reaction temperature and long synthesis time made AlN powder by CRN have a high cost although it had achieved large-scale commercialization. In addition, the detailed mechanism of the carbothermal reduction reaction between different raw materials was not fully understood. It was still common to see the reports improving the types of raw materials [11,12,13], using the suitable additives [14,15,16] and preparing the near-spherical AlN powder [17,18] in recent years.

In the CRN process, the reaction using Al_2_O_3_ and carbon as raw materials could be expressed by [19]

Al_2_O_3_(s) + 3C(s) + N_2_(g)→2AlN(s) + 3CO(g) (1)

Aluminum hydroxide (Al(OH)_3_) attracted more and more attention as one of the Al sources because of its higher nitridation reaction rate than that of the conventional α-Al_2_O_3_ [20,21,22,23]. The reaction chemical equation was similar to Al_2_O_3_ as follows:

2Al(OH)_3_(s) + 3C(s) + N_2_(g)→2AlN(s) + 3CO(g) + 3H_2_O(g) (2)

In the CRN process, the mixing uniformity between Al(OH)_3_ and carbon powder was the key factor to achieve the high nitridation degree and low agglomerate. In addition, the tap density of the mixed raw materials was also important affecting the yield in the certain reaction container. In the common mixing by ball-milling using water or organic solvents as dispersing media, the long drying time and loose stack of raw materials reduced the output and also increased the cost of the product. However, the study on the mixing process was seldom reported in the literature.

The three-roll mixing process with the advantages of simple operation, fast grinding speed and large dispersion strength was mainly used for the grinding of liquid pastes containing various paints, inks, pigments, plastics, cosmetics, soaps, ceramics, silicon liquid rubber, etc. [24,25]. More importantly, the mixing by three-roll method used a very small amount of dispersion medium different from the ball-milling. However, it cannot be directly used to mix the hard Al(OH)_3_/C system.

Polyvinyl butyral (PVB) resin was one of the most common binders in non-water-based casting systems due to its good adhesion, thermal decomposition and low residual carbon after being fired in the air [26,27]. In this paper, PVB–ethanol solution was added to the Al(OH)_3_/carbon black powders to make them suitable for three-roll mixing. The effects of PVB on the viscosities of the slurries, the stacking densities of the mixed raw material powders, the CRN process of AlN powder and the carbon removal process were further discussed.

## 2. Experimental

### 2.1. Experimental Procedure

**Figure 1:**
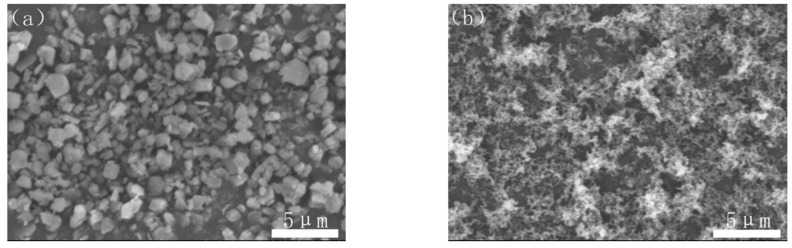
SEM images of (**a**) Al(OH)_3_ and (**b**) carbon black.

Commercially available Al(OH)_3_ powder (Chalco Shandong Co., Zibo, China, H-WF-1, 99.9% purity), carbon black powder (Hangzhou Juy New Materials Technology Co., Hangzhou, China, N124, 99.9% purity) and PVB (B-98) were used as the starting materials. The morphologies of Al(OH)_3_ ball-milled for 4 h at the rate of 250 r/min with the particle size of 0.5–2 μm and carbon black were observed as shown in Figure 1.

PVB was solved into 80 mL ethanol and then added to the mixed powders containing Al(OH)_3_ and carbon black with the mass ratio of 3:1 to achieve the slurries. The compositions of different samples with PVB additions were listed in Table 1.

The slurries were milled by the three-roll mill for 50 min and then dried at 80 °C. The dried powders were placed in the graphite furnace and fired at 1550 °C for 3 h under the flowing nitrogen at the rate of 0.2 L/min. Then, the calcined powders were fired under the drying air atmosphere at 700 °C for 3 h to remove the excess carbon black.

### 2.2. Characterization

The viscosities of the Al(OH)_3_/carbon/ethanol slurries at different PVB additions were measured using a rotational viscometer (Brookfield, Middleboro, U.S.A.) at 25 °C. The samples A–F after being milled and dried were placed in the standard graduated cylinder with 25 mL to achieve their tap densities according to the formula:

d_t_ = M/V (3)

Here, d_t_, M and V were the tap density, mass and volume of the powder, respectively. The phase compositions of the nitridized samples A–F were identified by X-ray diffraction (PANalytical, Almelo, The Netherlands) with Cu Kα1 radiation (λ = 0.154056 nm). The morphologies of the nitridized samples A–F were observed using scanning electron microscope (Hitachi, Tokyo, Japan). The final nitridized products (AlN powder) were ultrasonicated in ethanol for 20 min, and then the median diameter (D50) and particle size distribution were measured by laser particle size analyzer (Microtrac, Florida, U.S.A.). The TG analysis (TA Instruments, New Castle, U.S.A.) between 0 and 700 °C were carried out to detect the weight change of the single PVB, the sample C, G and H containing Al(OH)_3_ in the nitrogen atmosphere at the heating rate of 5 °C/min.

## 3. Results and Discussion

### 3.1. Viscosities of the Slurries

**Figure 2:**
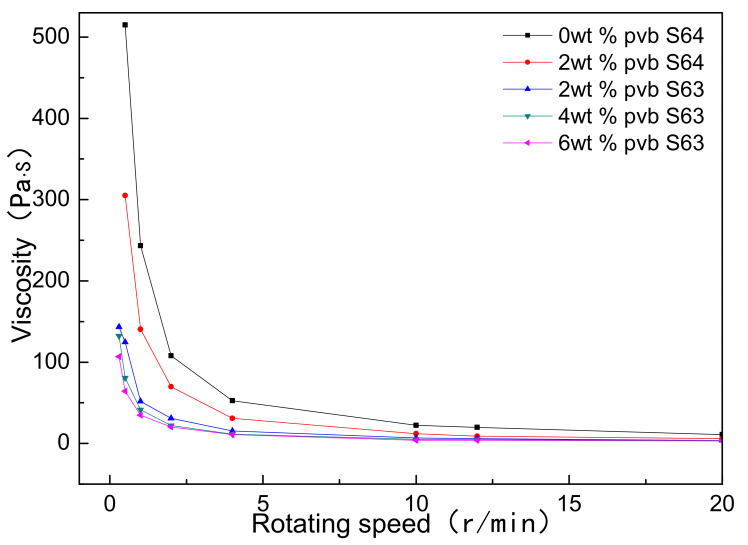
Viscosity changes of Al(OH)_3_/carbon/ethanol slurries with different PVB additions.

Figure 2 showed the viscosity changes of Al(OH)_3_/carbon/ethanol slurries with different contents of PVB. S63 and S64 were the rotor numbers of rotary viscometer. It could be seen that as the PVB additions increased, the viscosities of the Al(OH)_3_ and carbon black slurries were reduced, different from the common action of PVB as a binder. It was also found that the viscosities of the samples C and D were almost equal. The decrease in viscosity implied that the mixing homogeneity of Al(OH)_3_ and carbon black particles were improved under the same mixing conditions as when PVB was used.

### 3.2. Tap Densities of Al(OH)_3_/Carbon Black Powders

**Figure 3:**
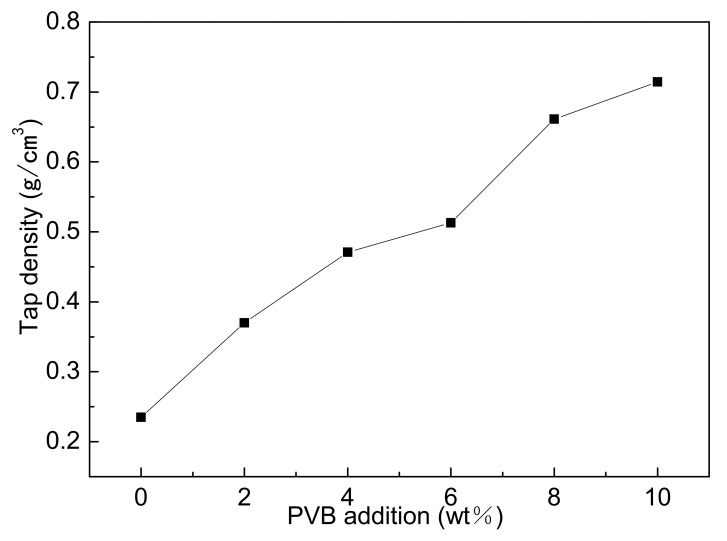
Tap densities of Al(OH)_3_/carbon black powders with different PVB additions.

Figure 3 showed the tap densities of different samples mixed by three-roll with different PVB additions. It could be seen that the tap densities gradually increased with the increase in0 PVB content. PVB covering the surface of Al(OH)_3_ and carbon black particles increased the compatibility between the powder particles during the mixing, thus narrowing the distance between particles and causing the increase in tap density after drying.

### 3.3. Morphologies of Al(OH)_3_/Carbon Black Powders

**Figure 4:**
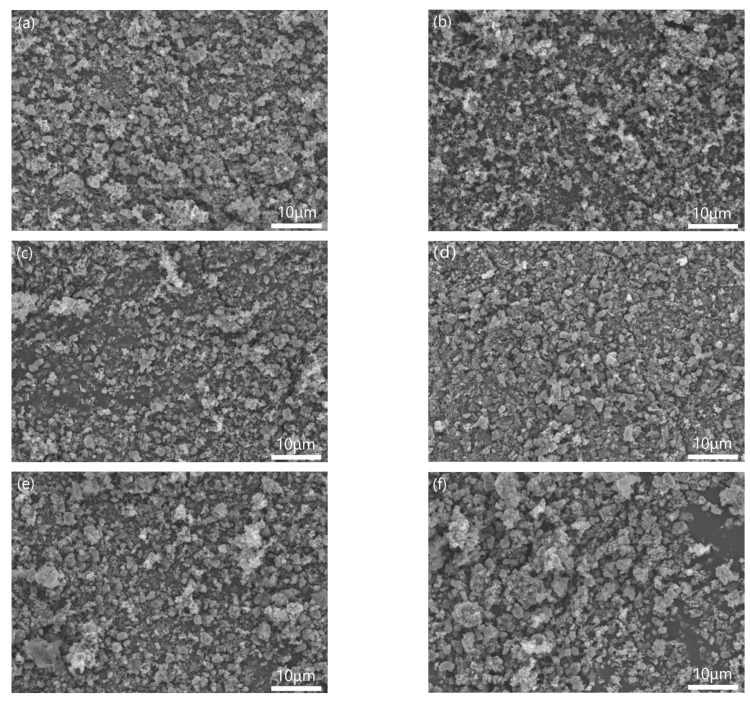
Morphologies of the mixed Al(OH)_3_/carbon black powders with different PVB additions: (**a**) sample A; (**b**) sample B; (**c**) sample C; (**d**) sample D; (**e**) sample E; and (**f**) sample F.

Figure 4 showed the morphologies of different samples mixed by three-roll with different PVB additions. When the PVB contents were low (Figure 4a,b), the Al(OH)_3_ powders agglomerated and could not be distributed sufficiently. Then, the agglomerated particles decreased with the increase in PVB contents (Figure 4c,d). When the PVB contents were up to 8 wt.% and 10 wt.% (Figure 4e,f), the large agglomerated particles with Al(OH)_3_ and carbon black bound by PVB were clearly seen. The increase in tap density shown in Figure 3 agreed with the distributing change in the mixed powders.

### 3.4. Phase Compositions of the CRN Products

**Figure 5:**
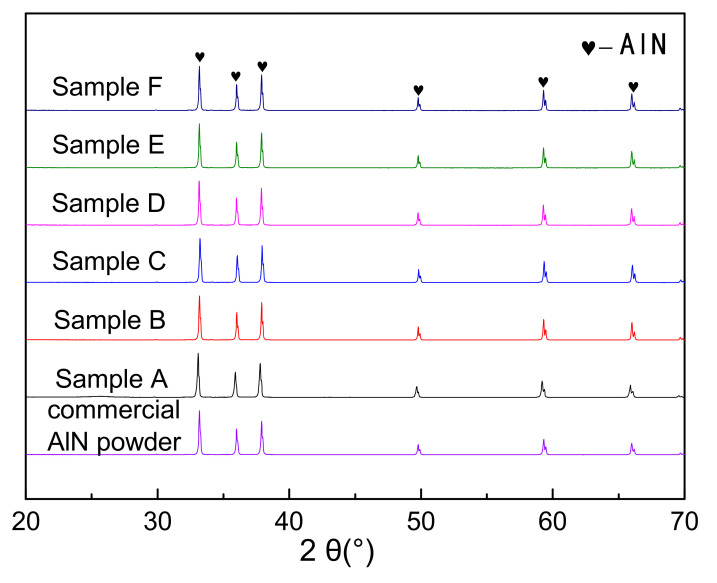
XRD patterns of the carbothermal reduction–nitridation (CRN) powders with different PVB additions and commercial AlN powder.

Figure 5 showed the X-ray diffraction patterns of the nitridized products with different PVB additions. The commercial AlN powder (Tokuyama) was also identified as a contrast. The nitridized products displayed the same peaks of AlN regardless of the PVB amounts, indicating that the raw powders were completely nitridized when the PVB weight ratio in Al(OH)_3_ and carbon black were in the range of 0–0.1. The HWHM (half width at half maximum) values at the highest peaks of the CRN AlN (samples A–F) were 0.158°, 0.146°, 0.150°, 0.152°, 0.146° and 0.146° respectively, which were similar to the commercial AlN powder (0.152°). Combining Figure 3, it was deduced that there was still sufficient nitrogen gas around the raw material powder to ensure the complete nitridation of alumina even though the tap density reached 0.71 g/cm^3^ (sample F).

### 3.5. Morphologies and Size Distributions of the CRN Products

**Figure 6:**
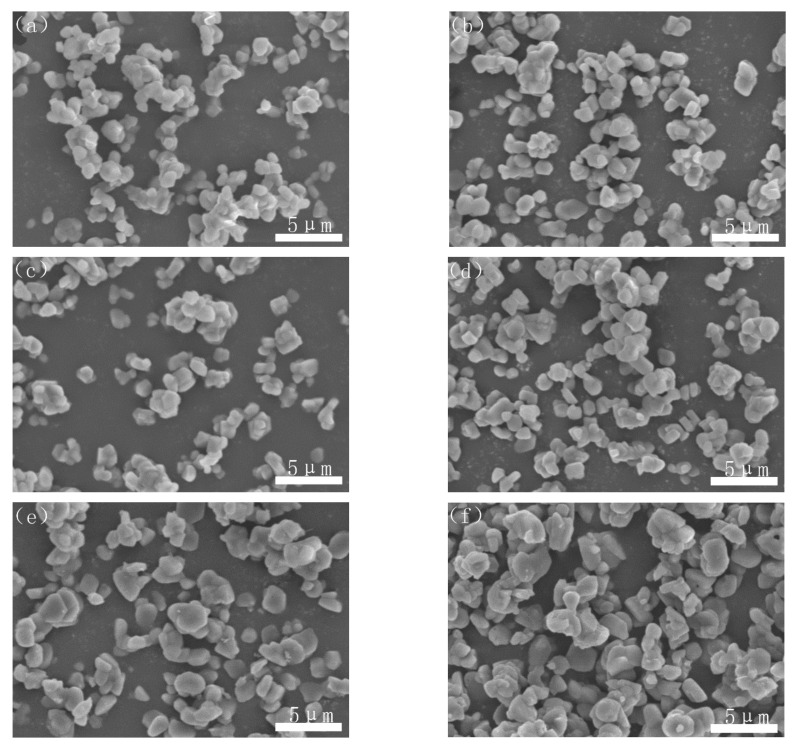
Morphologies of the nitridized powders with different PVB additions: (**a**) sample A; (**b**) sample B; (**c**) sample C; (**d**) sample D; (**e**) sample E; and (**f**) sample F.

Figure 6 showed the morphologies of the nitridized powders of samples A–F. It could be seen that the agglomeration of the AlN powder was obvious in the sample without PVB addition (Figure 6a). With the addition of PVB, the particle distribution of the nitridized powders became better and better. However, when the PVB addition was above 8 wt.%, the agglomeration became serious again (Figure 6e,f). The agglomeration change of the CRN products was consistent with that of the unreacted mixed samples (Figure 4).

**Figure 7:**
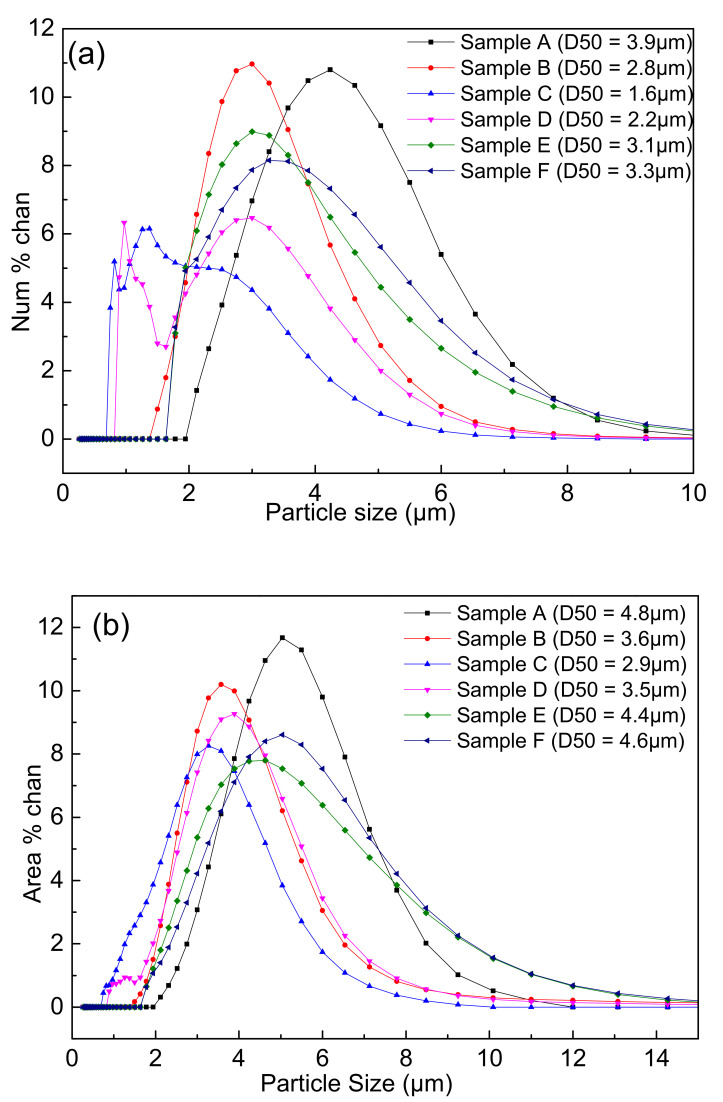
Particle size distributions of the nitridized powders with different PVB additions: (**a**) number distribution; and (**b**) area distribution.

Figure 7 showed the particle size distributions of the samples A–F after carbon removal. It could be seen that the peaks gradually shifted to the left with the increase in the PVB addition in the same three-roll mixing conditions. Thereafter, the peaks shifted to the right again after PVB weight ratio was 0.04 (sample C). It was strange that the two peaks occurred in the samples C and D, which may be associated with the existence of the fine particles with less agglomeration.

It was indicated from Figure 5 and Figure 6 that the effects of PVB on the agglomeration of the nitridized product came from two aspects. On the one hand, the Al(OH)_3_ and C mixtures became more homogeneous and dispersive with the increase in PVB additions, which would cause less agglomeration of the product powder. On the other hand, the tap density of the mixed powder became large and the distance between the Al(OH)_3_ particles was close due to the increase in PVB addition (Figure 3), which resulted in the serious agglomeration of the product particles. In the experiments, when PVB was added with the weigh ratios of 0.04 and 0.06, the degree of agglomeration of the product particle was the minimum, which may cause the occurrence of the additional peak of small size in Figure 7.

### 3.6. The Action Mechanism of PVB in CRN Process

**Figure 8:**
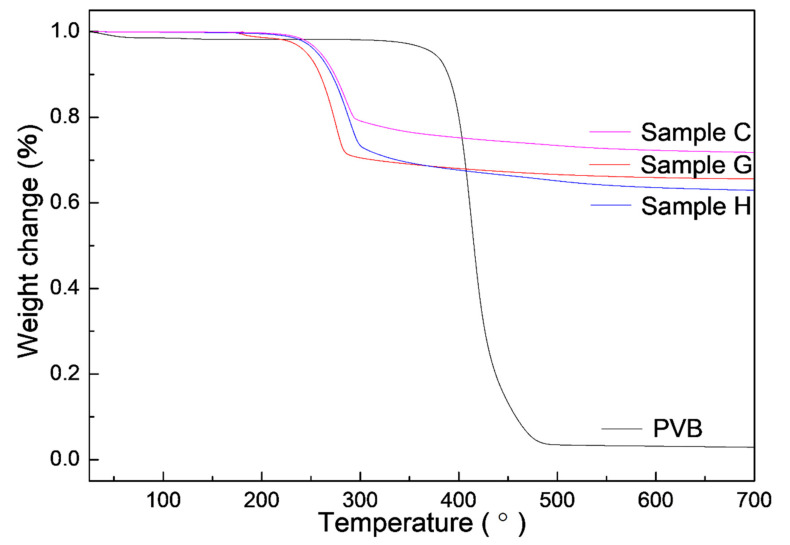
TG curves of the single PVB and samples G, H and C containing Al(OH)_3_.

As known, PVB was often used as a binder in the dry pressing and tape casting of AlN ceramics and could be burned out in air at 600 °C [28]. To further study the change of PVB in Al(OH)_3_ and carbon, the TG curves were measured by the single PVB, sample G (the single Al(OH)_3_), H (Al(OH)_3_/PVB) and C (Al(OH)_3_/carbon black/PVB) as shown in Figure 8. The weight changes at different temperatures were listed in Table 2. It could be seen that the single PVB had a sharp weight loss of 94.39% between 300 and 486 °C. When the temperature reached 700 °C, the whole weight loss of PVB was high to 97.11%. This indicated that most PVB evaporated in the nitrogen atmosphere. Differently, the main weight loss occurred between 200 and 300 °C in samples G, C and H containing Al(OH)_3_, which was associated with the elimination of the hydrate water in Al(OH)_3_. It could also be calculated that the weight loss of 34.390% in sample G was very close to the theoretical percentage of H_2_O in Al_2_O_3_⋅3H_2_O (34.62%). On the data of the single PVB and Al(OH)_3_, the calculated weight changes of samples H and C were consistent with the experimental value in every temperature range (i and ii in Table 2). This indicated that there was a similar weight loss of PVB in the single PVB, Al(OH)_3_/PVB and Al(OH)_3_/carbon/PVB systems. Since nearly all the PVB disappeared in the fired process, the nitrogen could have sufficient passages to finish the CRN reaction.

**Figure 9:**
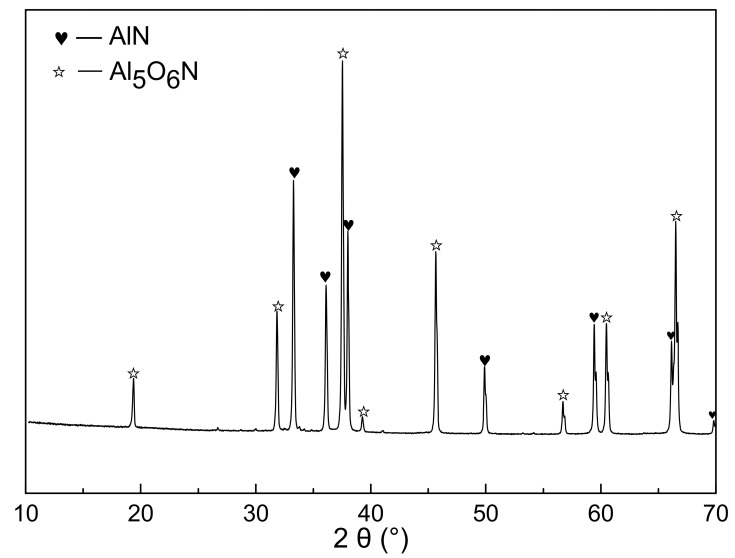
XRD patterns of fired powders with Al(OH)_3_ and PVB (sample I).

Figure 9 showed the XRD patterns of the nitridized products from sample I. The obvious diffraction peaks of AlN and aluminum oxynitride compound (Al_5_O_6_N) were observed. It was also noted in Figure 8 and Table 2 that about 3 wt.% PVB remained after being fired in the nitrogen. This implied that a small amount of carbon from the thermal decomposition of PVB in the nitrogen atmosphere still remained and acted in the CRN process when PVB was added into Al(OH)_3_ mixture.

**Figure 10:**
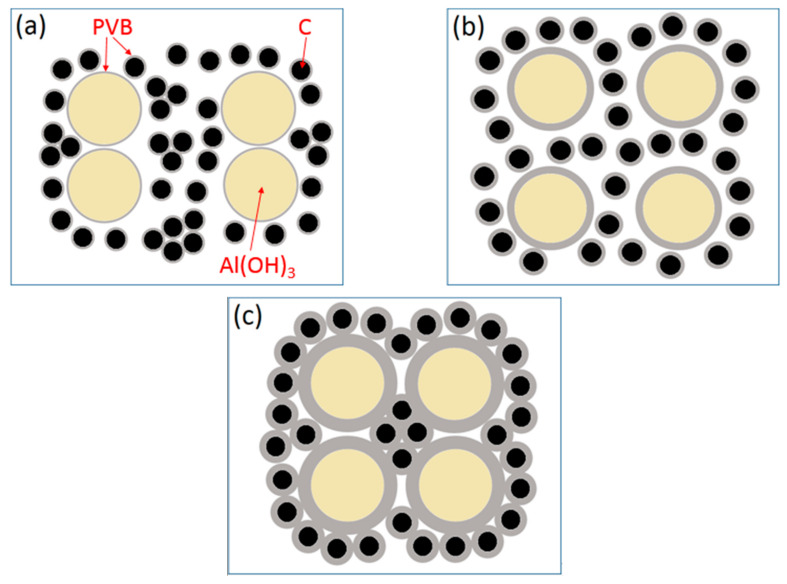
Sketch map of Al(OH)_3_/carbon black mixture covered with PVB layer: (**a**) thin PVB layer; (**b**) moderate PVB layer; and (**c**) thick PVB layer.

At the base of the experiments, the action mechanism and change of PVB were proposed to explain the results that the proper PVB contents benefited achieving the CRN powders with the single AlN phase and the good distribution in the situation of high tap density as shown in Figure 10. As seen in Figure 2, the viscosities of the Al(OH)_3_ and carbon black slurries were reduced with the increase in PVB additions up to 6 wt.%, which indicated that the mixing homogeneity of Al(OH)_3_ and carbon black particles were improved under the same mixing conditions due to the deformation and sliding of the PVB layer. After drying, the PVB formed a layer of binder covering the Al(OH)_3_ and carbon black particles. As shown in Figure 10a, when PVB was not used or used with small contents, it was difficult to distribute Al(OH)_3_ and carbon particles sufficiently, resulting in the serious agglomeration of final AlN product particles (Figure 6a,b). As the amounts of PVB increased, the Al(OH)_3_ particles were separated by the carbon particles and decreased the agglomeration (Figure 10b), which improved the distribution of product particles (Figure 6c) although the tap densities simultaneously increased (Figure 3). When PVB addition was too high, the distance of Al(OH)_3_/carbon black particles was shortened, which further increased the tap densities (Figure 3) like the ceramic granulation process before forming. Although the tap density increased with the increase in PVB content, most PVB disappeared in the following CRN process as shown in Figure 8. Thus, the close Al_2_O_3_ from Al(OH)_3_ decomposition was easy to combine together at the high temperature of 1550 °C and resulted in serious agglomeration when the carbon between them was completely reacted (Figure 10c).

## 4. Conclusions

The PVB addition obviously reduced the viscosity of Al(OH)_3_/carbon/ethanol slurry and promoted the distribution of Al(OH)_3_ and carbon black powders by three-roll mixing. At the same time, the tap densities of the Al(OH)_3_/carbon system were also increased from 0.24 g/cm^3^ (no PVB) to 0.71 g/cm^3^ (0.1 PVB weight ratio) with the similar AlN products after CRN reaction. With the increase in PVB addition, the agglomeration of the CRN AlN powder began to decrease and then became serious. The proper weight ratios of PVB in Al(OH)_3_ and carbon black with 0.04 and 0.06 benefited from decreasing the agglomeration and achieving the fine AlN particles. In the CRN process, most PVB covering Al(OH)_3_ particles evaporated and supplied the passage for the nitrogen removal to the particles, which made the CRN action possible in the case of the increasing tap density of the raw materials.

## Figures and Tables

**Table 1:** Compositions of the different samples with polyvinyl butyral (PVB) additions.

Sample	Al(OH)_3_ (g)	Carbon (g)	PVB (g)	m_(PVB)_/m_(Al(OH)3+Carbon)_ (wt. Ratio)
A	15	5	0	0
B	15	5	0.4	0.020
C	15	5	0.8	0.040
D	15	5	1.2	0.060
E	15	5	1.6	0.080
F	15	5	2.0	0.100
G	15	0	0	0
H	15	0	0.8	0.053
I	15	0	6	0.400

**Table 2:** The weight change (wt.%) of the single PVB, samples G, H and C at different temperature ranges.

T (°C)	PVB	Sample G	Sample H		Sample C	
			i	ii	i	ii
25–200	−1.759	−1.336	−0.477	−1.357	−0.288	−1.031
200–300	−0.031	−28.131	−26.118	−26.708	−20.544	−20.288
300–486	−94.393	−3.753	−7.903	−8.342	−5.514	−6.337
486–700	−0.925	−1.170	−2.569	−1.158	−1.865	−0.879
25–700	−97.108	−34.39	−37.067	−37.565	−28.211	−28.535

i: the weight change directly measured from the TG curve (Figure 7) of the samples; ii: the weight change calculated from the single PVB and Al(OH)_3_ (sample G) in Figure 7.

## Data Availability

The data presented in this study are available on request from the corresponding author.
